# Detection of MMTV-Like sequences in Moroccan breast cancer cases

**DOI:** 10.1186/1750-9378-9-37

**Published:** 2014-11-10

**Authors:** Meriem Slaoui, Mohammed El Mzibri, Rachid Razine, Zineb Qmichou, Mohammed Attaleb, Mariam Amrani

**Affiliations:** Equipe de recherche ONCOGYMA, University of Mohamed V-Souissi, Faculty of Medicine and Pharmacy of Rabat, Avenue Mohammed Belarbi El Alaoui – Souissi, BP 6203 Rabat, Morocco; Unité de Biologie et Recherche Médicale, Centre National de l’Energie, des Sciences et des Techniques Nucléaires, Rabat, Morocco; Laboratory of Biostatistics, Epidemiology and Clinical Research, Université Mohamed V-Souissi Faculty of Medicine and Pharmacy of Rabat, Avenue Mohammed Belarbi El Alaoui – Souissi, BP 6203 Rabat, Morocco; Department of Public Health, Université Mohamed V-Souissi Faculty of Medicine and Pharmacy of Rabat, Avenue Mohammed Belarbi El Alaoui – Souissi, BP 6203 Rabat, Morocco

**Keywords:** Breast cancer, MMTV-like, Morocco

## Abstract

**Introduction:**

The mouse mammary tumor virus (MMTV) like sequences have been reported to be present in some human breast cancers, but their association with breast cancer development is still controversial.

**Methods:**

In this retrospective study, we investigated the status of MMTV-like in 42 tumor biopsies and 18 paired normal tissues from Moroccan patients with breast cancer. MMTV-like *env* sequences were identified by PCR and confirmed by direct DNA sequencing.

**Results:**

Specific MMTV-like *env* sequences were found in 24 (57.14%) cases of breast carcinomas, and 6 (33.3%) cases of matched normal breast tissues. Comparison to sociologic and clinicopathological parameters showed no significant association between the presence of MMTV-like sequences and age, menopausal status, histological subtype, histological grade, tumor size and the expression of hormone receptors (estrogen ER and/or progesterone PgR) and Her 2. However, a significant correlation was found between MMTV-like presence and parity (*p* = 0.024).

**Conclusions:**

This present study confirms the presence of MMTV-like *env* sequences in breast cancer in Moroccan women, prompting further evaluation, on large sampling, to elucidate the probable causal roles of MMTV-like in breast cancer development.

## Background

Worldwide, breast cancer is the leading cancer in women with approximately1.38 million new cases per year and 458.000 deaths annually [1]. In Morocco, breast cancer is the most predominant cancer and represents a great public health problem, with an age-standardized incidence rate (ASR) of 49.2 per 100 000 women [2].

Nowadays, multiple factors are associated with an increased risk of breast cancer, including age, gender, ethnicity, past history of breast cancer, reproductive and hormonal factors, family history and genetic factors, exposure to ionizing radiation, and environmental and lifestyle factors [3]. A high estrogen level is suspected to increase breast cancer risk, and other hormones such as progesterone, androgen derived from the ovaries and adrenal, thyroid hormones and insulin may play important roles in breast cancer development [3]. However, it’s widely accepted that hereditary transmission of some predisposition genes, especially BRCA1 and BRCA2, are the most known factors to be directly involved in the pathogenesis of breast cancer and is associated with 5–10% of breast cancer cases [4].

Since the discovery of the role of mouse mammary tumor virus (MMTV) as the causal agent of mammary tumor in mice [5], the viral etiology of human breast cancer has gained a growing interest. DNA sequences showing homology to those of MMTV virus have been detected in human breast cancer, suggesting that this virus called MMTV-like, also called Human Mammary Tumor Virus (HMTV), could be the human form of the MMTV and may be involved in the development of human breast cancer [6–9].

More recently, several studies have demonstrated the presence of MMTV-like *env* sequences in 30 – 40% of breast cancer cases in several Western countries including the United States, Italy, Brazil and Argentina [10]. Overall, the prevalence of MMTV-like ranges between 78% in Australia to 0% in Iran, Mexico, Germany and Japan [11–15].

Thus, the present study is the first one to be undertaken to determine the presence of MMTV-like in a set of Moroccan breast cancer samples and to evaluate the association between MMTV-like infection and some clinicopathological parameters. The aim of this study is to evaluate the involvement of MMTV-like in the development of breast cancer.

## Methods

### Samples

In this retrospective study, 42 formalin fixed paraffin embedded samples were collected at the pathology department of the National Institute of Oncology in Rabat from patients with breast cancer. To evaluate the presence of MMTV-like in non cancerous tissues, paired normal tissues from 18 patients were also analyzed. For these cases, sampling was done at least 2 cm away from the tumor. No consent was needed for this retrospective study. The study protocol was approved by the Ethical Committees of the Faculty of Medicine and Pharmacy of Rabat.

### Clinicopathological parameters

All the data were retrieved from patients’ medical files, including age, parity, menopausal status, histological type and grade, tumor size, hormonal status and Her2 assessment.

Hormonal receptors (ER, PgR) were considered positive when ER and /or PgR were positive. The positive cut off is nuclear staining in at least 10% of tumor cells with any intensity.

### DNA extraction

Sections of paraffin blocks were treated first with xylene to dissolve the paraffin and with ethanol to remove the remaining xylene. Cells were then lysed in the digestion buffer (Tris–HCl pH 8.0 10 mM, EDTA 10 mM, NaCl150 mM and SDS 2%) containing proteinase K (0.1 mg/ml). DNA isolation was performed with phenol-chloroforme extraction and ethanol precipitation. DNA was then resuspended in sterile distilled water [16]. The tissue DNA was precipitated with 2/5 volumes of 7.4 M ammonium acetate and 2 volumes of 100% ethanol, followed by incubation at -20°C and centrifugation at top speed (13000 relative centrifugal force). DNA was then resuspended in 25–30 μL of sterile distilled water and stored at -20°C until use. In order to evaluate the efficiency of DNA extraction, all samples were amplified by Polymerase Chain Reaction (PCR) using PC04 and GH20 primers specific for human β-globin gene (Table 1).

**Table 1:** **List of primers used for PCR amplification and DNA sequencing**

	Primer	Fragment generated size	Sequence	Tm (°C)
β-globin	PC04	268 bp	5′-CAACTTCATCCACGTTCACC-3′	54.5
	GH20		5′-GAAGAGCCAAGGACAGGTAC-3′	
MMTV-like detection	489 F*	171 bp	5′-ACCAGGGGGTGAGTTTTTCT-3′	57
	659R*		5′-CCCATCCTGCYTCATACCAT-3′	

*F: Forward primer; *R: Reverse primer.

### Detection of MMTV-like sequences by PCR

MMTV-like detection was performed by PCR using MMTV659R/MMTV489F primers (Table 1). These primers amplify a fragment of 171 bp of MMTV-like highly conserved in the *env* gene encoding the viral coat protein.

Amplification reaction was performed in a total volume of 25 μl. The amplification mixture contained 0.4 μM of each primer, 200 μM of each dNTP (dATP, dCTP, dGTP and dUTP), 0.5 units *Taq* DNA polymerase (Promega, France) and 3 μl of DNA sample in 1× *Taq* polymerase buffer. For every reaction, a negative control in which DNA template was omitted from the amplification mixture, and a positive control (MMTV DNA) were included. The thermal cycler (Gene Amp, PCR system 9700, Applied Biosystem, Foster city, CA) was programmed for 40 cycles with an initial denaturation at 94°C for 4 min. Each cycle was performed with a denaturation at 94°C for 30 sec, annealing at 57°C for 1 min and extension at 72°C for 1 min 30 sec. At the end of the last cycle, the mixtures were incubated at 72°C for 10 min.

The amplified products were submitted to electrophoresis on a 2% agarose gel in 1× Tris-borate-EDTA buffer at pH of 8.6. The gel was stained with ethidium bromide of 10 mg/ml 2 μL in 100 mL of 1× Tris-borate-EDTA, and the 171-bp amplified bands were visualized on an ultraviolet transilluminator to check for DNA amplification.

### DNA sequencing

For each PCR product, both strands were sequenced, in independent reactions, using MMTV659R/MMTV489F primers. Firstly, the PCR products were purified by the ExoSaP-IT clean up system (USB, USA).Direct sequencing was performed on an ABI 3130XL DNA analyzer (Applied Biosystems, Foster city, CA, USA), using the sequencing primer and BigDye^®^ Terminator v3.1 Cycle Sequencing Kit (Applied Biosystems, Foster city, CA, USA), according to manufacturer’s protocol. Nucleotides sequences were aligned and compared to the reference sequence of MMTV-like (Accession number: AF243039) and MMTV (Accession number: AY152721).

### Statistical analysis

Categorical variables were expressed as numbers and percentages, and continuous variables were expressed as means ± SD or median (interquartile range). Data were analyzed using the statistical soft-ware SPSS version 13.0.

The results were compared using the Chi2 test to identify associations between the different collected parameters and MMTV-like status. The statistical relationship was considered as significant if the derived p value was < 0.05.

## Results and discussion

### Results

The mean age of patients was 41.6 [36.00 – 49.25] years, with extreme ages at 27 and 73. Among the 42 recruited patients, 29 were diagnosed having Inflammatory Breast Cancer (IBC) (69%) while the remaining 13 cases were Non Inflammatory Breast Cancer (NIBC) (31%). Interestingly, more than 78% of the patients were premenopausal and 31.71% were nulliparous.

The pathological results of the 42 breast cancer cases are reported in Table 2. Except for one patient who had Invasive Lobular Carcinoma (ILC), all the patients (97.62%) were diagnosed with Invasive Ductal Carcinoma (IDC).

**Table 2:** **Pathological Characteristics of the 42 breast cancer cases**

Characteristics		Number of cases (%)
**Menopausal status**	Pre-menopausal	33 (78.57)
	Post-menopausal	9 (21.43)
**Histological type**	Invasive ductal carcinoma	41 (97.62)
	Invasive lobular carcinoma	1 ( 2.38)
**Histological grade**	I	3 ( 7.14)
	II	18 (42.86)
	III	21 (51.22)
**Tumor size**	≤20 mm	3 ( 7.14)
	20-50 mm	18 (41.86)
	>50 mm	21 (51.22)

Histopathologicalgrade II and III were the most frequent with a large tumor size in half of the cases (Table 2).

Immunohistochemistry analysis showed that hormone receptors were positive in 78.57%. The human epidermal growth factor receptor 2 (Her2) status was positive in 28.57% of the cases (Table 3).

**Table 3:** **Hormone receptors and Her2 results of the 42 breast cancer cases**

Marker		Number of cases (Percentage)
**ER**	Positive	19 (45.24)
	Negative	23 (54.76)
**PgR**	Positive	32 (76.19)
	Negative	10 (23.81)
**Her2**	Positive	12 (28.57)
	Negative	30 (71.43)

The presence of amplifiable DNA was confirmed for all 42 cases by PCR based-technique using primers for a fragment of *β-globin* gene and therefore all DNA samples were adequate for further analysis. Molecular detection of MMTV-like DNA, using PCR amplification of a conserved region of the viral *env* gene DNA, revealed the presence of viral DNA in 57.14% of breast cancer cases (24 of 42).

MMTV-like detection was also performed on 18 normal tissues anatomically related to tumor tissues, from patients with breast cancer. Results showed that 33.3% of cases are MMTV-like positive (6/18). Among them, 3 cases have MMTV-like *env* sequences in both normal and tumoral tissues whereas 3 cases have shown the absence of viral sequences in tumoral tissues.

The comparison of the clinicopathological and immunohistochemical data between the MMTV-like positive and MMTV-like negative breast cancer cases is summarized in Table 4. Overall, no association was found between the presence of MMTV-like sequences and age, menopausal status, the histological type, the SBR grade and the tumor size. Moreover, immunohistochemical expression of hormone receptors and Her2 did not vary significantly between MMTV-like positive and MMTV-like negative samples. However, our results clearly showed asignificant correlation between the presence of MMTV-like sequences and parity (P = 0,024).

**Table 4:** **Correlation of MMTV-like status according to the clinicopathological parameters of breast cancer cases**

Variables	N	Positive MMTV-like samples (%)	Negative MMTV-like samples (%)	P-value
Age				
<35 years	8	2 (25)	6 (75)	0,093
35-50 years	26	16 (61.5)	10(38.5)	
>50 years	8	6 (75)	2 (25)	
Nulliparity				
Yes	13	4 (30.8)	9 (69.2)	0,024
No	29	20 (69)	9 (31)	
Menopausal status				
Pre-menopausal	35	19 (54.3)	16 (45.7)	0,344
Post-menopausal	7	5 (71.4)	2 (28.6)	
Inflammatory breast cancer				
Yes	29	19 (65.5)	10 (34.5)	0,097
No	13	5 (38.5)	8 (61.5)	
SBR grade				
I	2	1 (50.0)	1 (50)	0,762
II	23	12 (52.2)	11 (47.8)	
III	17	11 (64.7)	6 (35.3)	
Tumor size				
≤20 mm	3	3 (100)	0 (0)	0,348
20-50 mm	18	9 (50)	9 (50)	
>50 mm	21	12 (57)	9 (43)	
ER				
+	19	9 (47.4)	10 (52.6)	0,349
-	23	15 (65.2)	8 (34.8)	
PgR				
+	32	20 (62.5)	12 (37.5)	0,281
-	10	4 (40)	6 (60)	
Her2				
+	12	7 (58.3)	5(41.7)	0,600
-	30	17 (56.7)	13(43.3)	

**Figure 1:**
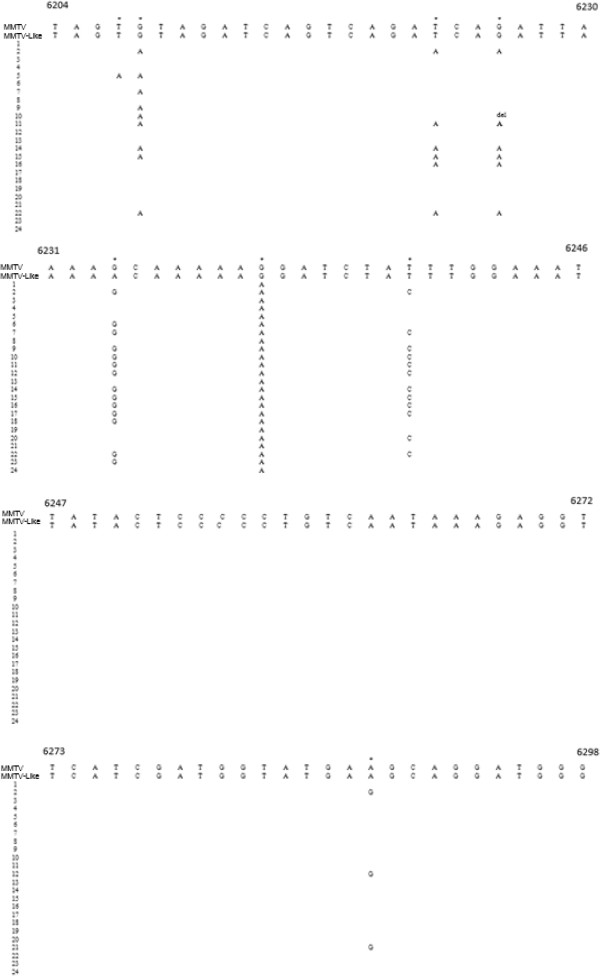
**Multiple nucleotide alignment of the MMTV-like** ***env*** **sequences of 24 human breast carcinomas with the MMTV C3H (GenBank: AF228552.1) and MMTV-like SAG pseudogene (GenBank AF243039.1) reference sequences.**

All samples identified as positive for MMTV-like *env* sequence were sequenced in order to confirm the presence of MMTV-like in breast cancer samples. The complete PCR products’ sequences, aligned to MMTV and MMTV-like sequences are reported in Figure 1. Multiple nucleotide alignment showed 95-99% homology to MMTV and MMTV-like *env* sequences.

Overall, 8 point mutations and 1 deletion were found when comparing obtained sequences to the MMTV-like reference sequence. Interestingly, the point mutation G6241A is present in all analysed samples. Moreover, 10 of 24 cases have a nucleotide A at position 623, as the MMTV-like sequence, whereas the remaining 14 cases had a G, as the MMTV sequence.

## Discussion

Worldwide, the viral etiology of breast cancer is largely discussed and remains controversial. During lasts decades, there is a resurgence of interest in a potential role of MMTV-like, a retrovirus homologue of MMTV involved in breast cancer in mice [17].

Many studies have reported that sequences highly homologous to MMTV were found in up of 40% of human breast cancers [18]. To our knowledge, this study is the first one conducted in Morocco to assess the involvement of MMTV-like in the development of breast cancer.

In our current study, the viral sequences were detected in 57.14% of cases. Additionally, the MMTV-like env sequences detected in our cases were highly homologous to the MMTV and the MMTV-like sequences reported previously [19, 20]. MMTV-like detection in African countries is limited to the Tunisian population as reported by Levine et al. [21] and Hachana et al. [22]. The breast cancer samples analysed by Levine et al. [21] were characterized by the predominance of the inflammatory form and showed a high frequency of MMTV-like (74%). However, in the study of Hachana et al. [22], inflammatory breast carcinoma was found only in 1.6% of cases (2/122), and only 14% of cases were MMTV-like positives. This difference suggested a correlation between MMTV-like and inflammatory breast cancer. In our study, MMTV-like was found in both inflammatory and non inflammatory breast cancer cases.

The association of MMTV-like and the inflammatory form of breast cancer was developed in a study of inflammatory breast cancer in the United States evaluating biospecimens obtained from patients enrolled in the North American inflammatory breast cancer registry. Reported data clearly showed that 71% of inflammatory breast cases were MMTV-like positives, as compared with 40% of non-inflammatory breast cancer cases (P < .0001) [23].

In addition, there were major geographic differences. In low incidence countries of breast cancer, MMTV-like *env* sequences were detected in less than 17% of cases. Indeed, studies of breast cancer cases from Japan and China have detected MMTV-like *env* sequences respectively in 12% [24] and 16.8% of tumors [25].In contrast, our result concords with the other studies performed in high-incidence countries of breast cancer, such as the United States where MMTV-like sequences were identified in 40–70% of breast cancer cases [23]. Similarly, studies of breast cancer cases from Argentina have detected MMTV-like sequences in 31.7% of the tumors [26, 27]. Interestingly, Mazzanti *et al*. have reported the presence of MMTV-like *env* sequence in 82% of ductal carcinoma *in situ*, but only in 35% of infiltrating ductal carcinoma [28]. In Australia, many studies have been conducted and showed a large discrepancy. Ford *et al*. [9] have reported MMTV-like sequences in 40% of breast cancer cases, but more recently a study conducted by Glenn et al. [29] revealed the presence of MMTV-like in 78% of the cases which is the highest frequency published up to now.

On the other hand, other studies conducted in England, Iran, Germany and Mexico, have never found the MMTV-like sequences in their breast cancer samples [30, 12–14].

The observed differences could be explained by the technique used in the detection of the MMTV-like sequences; the quality of DNA obtained from breast cancer biopsies and could also be related to the prevalence of *Musdomesticus* in these regions. In fact, it’s widely accepted that mice might act as a reservoir and transmit the virus to humans [17]. Moreover, human breast cancer is higher in geographic areas (e.g., Western Europe, USA) where *Mus domesticus* is the most prevalent mouse species than other regions (e.g., Asia) and that *M. domesticus* mice produce more MMTV as they carry more exogenous virus and have more endogenous proviral loci than *M. musculus*[17].

In this study, MMTV-like sequences were found in 33.3% of normal tissues sampled at least 2 cm away from the tumor. Many studies have investigated the presence of MMTV DNA in normal tissues and showed the presence of MMTV-like *env* sequences in 0-19% [9, 22, 28]. The presence of viral DNA in normal tissues was largely discussed and all converge to the exogenous origin of the virus infection [17].Moreover, the high frequency of MMTV-like *env* sequences in our cases could be due to the sensitivity of the technique of detection. Indeed, the nested PCR used in our study, which is more sensitive than PCR, is often needed to detect the presence of virus DNA, because of low-level infections in humans, in contrast to mice [17].

Interestingly, MMTV-like *env* sequences were detected in normal tissues from 3 patients with breast cancer and not in the paired tumoral tissues. For these cases, breast cancer could have another origin and patients were on-infected by the virus. Therefore, it will be interesting to follow-up closely these patients to evaluate the outcome of the disease in the presence of the virus.

In the present study, the analysis of clinicopathological parameters showed no significant correlation between the presence of MMTV-like sequences and histological subtype, histological grade, tumor size, hormone receptors and Her 2 expression, suggesting that the infection by the MMTV-like is not associated with the tumor subgroups examined and the evolution of the disease. According to age, and even if it’s not statistically significant, the viral DNA is more frequent in elderly women leading to a trend association between viral infection and patients age, suggesting that MMTV-like may be a contributing factor in human breast carcinogenesis and breast cancer related MMTV-like requires more time for cancer development.

However, a significant association was found between MMTV-like positive samples and parity. This could be explained by the modification of breast tissues during pregnancy and breastfeeding. Indeed, the breast tissues are subject to continuous transformation. Thus, after pregnancy the breast tissue could be more susceptible to viral infection [31].

## Conclusion

In conclusion, our study clearly shows the presence of MMTV-like *env* sequence in breast cancer cases in Morocco. This study prompts to conduct further evaluations, on large sampling, to elucidate the probable causal roles of MMTV-like in breast cancer development.

## Abbreviations

BC: Breast cancer
MMTV: Mouse mammary tumor virus
ASR: Age-standardized incidence rate
HMTV: Human mammary tumor virus
PCR: Polymerase chain reaction
IBC: Inflammatory breast cancer
NIBC: Non inflammatory breast cancer
ILC: Invasive LobularCarcinoma
IDC: Invasive DuctalCarcinoma
PgR: Progesterone receptor
ER: Estrogen receptor
Her2: Human epidermal growth factor receptor 2.
